# Richness and composition of Coleoptera (Curculionidae, Scarabaeidae and Passalidae) in a tropical coastal mountain range in Colombia

**DOI:** 10.3897/BDJ.14.e205516

**Published:** 2026-11-06

**Authors:** Ana Maestre-Guerra, Evelin Villalba-Fuentes, Hermes Villafaña-Márquez, Rubiel Chaparro Izquierdo, Andrés Redondo, Mayelis Barros-Barrios, Jennifer C. Girón Duque, Fernando Z. Vaz-de-Mello, Larry Jimenez-Ferbans

**Affiliations:** 1 Laboratorio de Biodiversidad, Sistemática y Biogeografía. Universidad del Magdalena, Santa Marta, Colombia Laboratorio de Biodiversidad, Sistemática y Biogeografía. Universidad del Magdalena Santa Marta Colombia; 2 Universidad del Magdalena, Santa Marta, Colombia Universidad del Magdalena Santa Marta Colombia; 3 Parques Nacionales Naturales de Colombia, Santa Marta, Colombia Parques Nacionales Naturales de Colombia Santa Marta Colombia; 4 Natural Science Research Laboratory, Lubbock, United States of America Natural Science Research Laboratory Lubbock United States of America; 5 Universidade Federal de Mato Grosso, Cuiabá, Brazil, and a member INCol - Instituto Nacional de Coleoptera - CNPq and CNPq fellow, Cuiaba, Brazil Universidade Federal de Mato Grosso, Cuiabá, Brazil, and a member INCol - Instituto Nacional de Coleoptera - CNPq and CNPq fellow Cuiaba Brazil

**Keywords:** altitudinal gradient, insect diversity, occurrence data, participatory research

## Abstract

**Background:**

Current conservation challenges have made it imperative to document insect fauna, which faces many threats, especially in megadiverse countries like Colombia. However, exploring large taxonomic groups at a regional scale imposes technical and budgetary limitations that are difficult to overcome. In this project, academics and members of the Arhuaca indigenous community of the Sierra Nevada de Santa Marta (SNSM), in Colombia, joined forces to study three groups of beetles (Coleoptera): weevils (family Curculionidae), bess beetles (family Passalidae) and dung beetles (Scarabaeidae, Scarabaeinae). These taxa have contrasting life histories and high ecological relevance constituting potential bioindicator organisms to track conservation status, anthropogenic disturbance and ecological changes.

**New information:**

The composition of two families and one subfamily of beetles was studied in two hydrographic basins of the SNSM, comprising nine sampling stations at elevations of 0–2000 m above sea level (m a.s.l.). For the family Curculionidae, 288 records of 666 individual belonging to 12 subfamilies, 35 tribes, 57 genera and 110 putative species (morphospecies) were recorded. All morphoespecies were assigned to subfamily, 99 to tribe and 97 to genus. The genera *Conotrachelus* Dejean, 1835 (Molytinae), *Geraeus* Pascoe, 1889 (Baridinae), *Anchonus* Schönherr, 1825 (Molytinae) and *Anones* Casey, 1922 (Baridinae) are represented by the highest numbers of species. The genera *Penestes* Schönherr, 1825 (Brachycerinae), *Plectrophoroides* Wibmer & O’Brien, 1986 (Entiminae) and *Cossonus* Clairville, 1798 (Cossoninae) accounted for 54% of the abundance. In Passalidae, 240 records of 12 species (four endemic) were found. In this case, all 1035 specimens were identified to the species level. *Passalus* Fabricius was the richest and most dominant genus, with seven species and 635 individuals. Finally, for Scarabaeinae, a total of 2435 records comprising 11885 individuals were obtained, representing 15 genera and 52 species, with all the records identified at least at the genus level. *Onthophagus* Latreille, 1802, *Uroxys* Westwood, 1842 and *Canthidium* Erichson, 1847 were the most abundant genera, together accounting for 85% of the individuals, whereas *Canthon* Hoffmannsegg, 1817, *Onthophagus* Latreille, 1802 and *Canthidium* were the most species-rich, representing 55% of the recorded species.

## Introduction

Guaranteeing the medium and long term environmental sustainability of ecosystems requires knowing their biological diversity at different scales. The impact of anthropogenic activities (e.g. agricultural and urban expansion) on biodiversity is more evident and drastic at landscape and regional scales (Kattan and Naranjo 2008); therefore, it is important to develop inventories at a regional scale. However, this is not easy, especially in megadiverse countries like Colombia, where ecosystem complexity and limited economic resources combine. In this scenario, the study of groups with contrasting life histories and high ecological relevance should be prioritised, so that they can be used as bioindicator organisms for conservation status, anthropogenic disturbance and ecological change, amongst others.

Amongst animals, insects are the most diverse group, with a little more than a million described species (Stork 2018) and, compared to vertebrates, they can be studied through sampling schemes that are easy to implement and offer a large amount of information in a relatively short time. In turn, within insects, beetles are the most specious group with close to 400,000 species (Bocak et al. 2013). Beetles participate in a wide range of ecological processes in aquatic and terrestrial environments and provide important ecosystem services, such as pollination, nutrient cycling and pest control. For instance, weevils in the family Curculionidae represent one of the main groups of herbivores in terrestrial ecosystems, since they are the second most species-rich family in the entire animal kingdom: 51,000 (Oberprieler et al. 2007). Bess beetles (family Passalidae) are a subsocial group that establishes colonies inside rotting logs, from which they feed, fulfilling the role of wood decomposers in tropical and temperate forests. Finally, dung beetles in the subfamily Scarabaeinae (family Scarabaeidae) have been widely used as a bio-indicator group to evaluate biodiversity and land-use impacts (Arellano et al. 2023), due to its intimate association with the excrement of vertebrates. These three families fulfil different, but equally important ecological roles and have been widely used in studies for assessing habitat quality, degree of ecosystem conservation and the establishment of protected areas (Schuster et al. 2000, Castillo and Lobo 2004, Schuster and Cano 2006, Beza-Beza et al. 2011, Majzlan et al. 2021, Haran et al. 2023, Cupello et al. 2023), highlighting the relevance of conducting comprehensive inventories of their diversity as a key step towards understanding ecosystem functioning and for supporting conservation planning.

The Sierra Nevada de Santa Marta (SNSM) is the highest coastal mountain system in the world (Parra et al. 2020), its highest peaks (5,775 m a.s.l) being located only about 50 km from the Caribbean Coast. Its pyramidal massif covers approximately 13,700 km² and has a complex geological history: its Cretaceous oceanic basement originated in an intra-oceanic arc that was accreted to the South American margin during the Palaeocene, temporarily integrating with the Central Andes before being displaced laterally (eastwards) in the late Eocene (Tschanz et al. 1974, Cardona et al. 2011, Parra et al. 2020). Likewise, owing to its broad altitudinal gradient and the full range of altitudinal zones present in Colombia, the SNSM harbours a remarkable diversity of ecosystems and is recognised as a major reservoir of biodiversity. It was designated an UNESCO Biosphere Reserve in 1979 (ProSierra 1998) and constitutes one of Colombia’s seven biogeographic provinces (Hernández-Camacho et al. 1992). The massif is widely recognised as a centre of endemism (e.g. 164 endemic plant species; Fortier et al. (2025)) and has been described as the most irreplaceable natural reserve in the world (Duran-Izquierdo and Olivero-Verbel 2021). Its marked ecological differentiation between lowland areas — biogeographically related to the Caribbean plains — and high-elevation zones, which harbour numerous endemic species in both fauna and flora, reflects its long-term geographic isolation from climatically similar regions such as the Andes, a condition that has promoted diversification and speciation processes (Hernández-Camacho et al. 1992, Carbonó and Lozano 1997).

Despite its recognition as a global biodiversity hotspot, the Sierra Nevada de Santa Marta (SNSM) faces severe conservation threats driven by accelerating deforestation, agricultural expansion, cattle ranching and climate-induced environmental changes (Etter et al. 2006, Duran-Izquierdo and Olivero-Verbel 2021). These anthropogenic pressures threaten unique habitats before their biological communities have been adequately documented, a problem exacerbated by the critical lack of systematic baseline inventories. Consequently, biodiversity conservation in the SNSM faces multiple, interrelated challenges, including insufficient faunal and floral inventories, lack of long-term monitoring, rapid habitat loss (including the loss of vegetation and glacial cover) and a notable scarcity of comprehensive studies addressing biodiversity across multiple taxonomic groups. This study presents the results of the first scientific expedition conducted along two rivers on the northern and western slopes of the SNSM, documenting the richness and composition of Curculionidae, Passalidae and Scarabaeinae and providing a baseline inventory of these groups along the altitudinal gradient. Fieldwork was carried out in collaboration with the Arhuaco Indigenous community, whose support enabled access to previously unsampled localities and facilitated a more comprehensive assessment of the region’s fauna. Thus, publishing these standardised occurrence records in an open-access format also ensures that biodiversity data return to the territory, serving as a tool for the Arhuaco community to document their natural heritage, monitor environmental health and strengthen legal and environmental arguments for the protection of their ancestral lands.

## General description

### Purpose

This inventory project aims to document the diversity of three families of beetles (Coleoptera: Curculionidae, Passalidae and Scarabaeidae: Scarabaeinae) in the northern and western slopes of the Sierra Nevada de Santa Marta.

## Project description

### Title

Diversity of insects and vertebrates, biosounds and ethnobiology on the northern and western slopes of the Sierra Nevada de Santa Marta.

### Personnel

The execution of this project relied on a participatory research framework established through active dialogue and co-design with members of the Arhuaco Indigenous community (including co-authors HVM and RCI). Prior informed consent and field logistics were coordinated with political and traditional indigenous authorities at each locality. Arhuaco community members participated directly in sampling expeditions across, sharing ecological knowledge regarding forest types, local microhabitats and terrain navigation. This collaborative approach facilitated safe access to sensitive and culturally restricted territories (such as high-elevation ancestral settlements in the Fundación and Don Diego River Basins).

### Study area description

The Sierra Nevada de Santa Marta is a pyramidal mountain massif, which reaches 5,775 m a.s.l. and which, at its base, covers an approximate area of 12,000 km² (Alvear et al. 2015). Its northern flank is more humid, presenting low humid forests, while the eastern flank is a little drier (Hernández-Camacho et al. 1992). The Fundación River Basin is located on the western flank, between 10°44'N – 10°17'N and 74°29'W – 73°50'W, occupying an area of 1,907 km^2^; the Don Diego River Basin is located between the coordinates 11°15' – 10°51' N and 73°37' – 73°52' W, with a total area of 561.95 km^2^.

### Design description

The study was carried out in nine landscape units (plots), located in the basins of the Fundación (western flank) and Don Diego (northern flank) rivers. In the Fundación River, six plots were studied at 0, 300-400, 800, 1,200, 1,600 and 2,000 m a.s.l.; in the case of the Don Diego River, three plots were established at 0, 400 and 800 m a.s.l. Each landscape unit covered an area of 1 km^2^, within which we attempted to incorporate the main types of vegetation cover, always ensuring that areas with native vegetation are included.

### Funding

The project was funded by Ministerio de Ciencia Tecnología e Innovación de Colombia through Patrimonio Autónomo Fondo Nacional de Financiamiento para la Ciencia, la Tecnología y la innovación Francisco José de Caldas (contract No. 80740-470-2021) and the Vicerrectoría de Investigación, Universidad del Magdalena.

## Sampling methods

### Study extent

Sampling events were carried out from June to December 2022.

### Sampling description

In each landscape unit, specimens from the groups under study were collected according to the following protocols:

**Curculionidae**. Specimens were collected using manual capture and entomological nets in free sampling walks. All captured specimens were deposited in vials containing 96% ethanol;

**Passalidae**. Rotten logs along free sampling walks were examined, these being completely destroyed using axes. Entire colonies were collected and deposited in jars with 96% ethanol;

**Scarabaeinae**. In each plot, 30 pitfall traps separated by 30 m were installed along two transects separated by at least 250 m. Traps were alternately baited with human excrement and rotting fish, so that, in each plot, there were 15 traps for each bait, that were left operating for 48 h. The traps consisted of a 500 ml plastic cup filled to one third with water and detergent. At the top, an inverted L-shaped wire was adapted to support a 25 ml cup with 35 g of bait.

### Quality control

Specimens were sorted by recognising distinct morphospecies. Identifications were made using specialised keys (Marvaldi and Lanteri 2005, Vaz-de-Mello et al. 2011, Jiménez-Ferbans et al. 2023, Arias-Buriticá and Vaz-de-Mello 2025) and by comparison with reference material deposited in the biological collections of the Universidad del Magdalena (Santa Marta, Colombia) and Instituto Alexander von Humboldt (Villa de Leyva, Boyacá, Colombia). To refine identifications, high resolution photographs of a specimen of each morphospecies were taken and sent to specialists including Robert Anderson (Canadian Museum of Nature, Canada; Curculionidae) and Arturo González (Universidade Federal de Mato Grosso, Brazil; Scarabaeinae), who provided identification at the lowest taxonomic category possible using images. Scarabaeinae identifications were directly evaluated and verified by co-author Fernando Z. Vaz-de-Mello, who has conducted extensive taxonomic revisions and examined primary type material for most South American dung beetle species. Taxa designated at the genus level (e.g. Canthon sp. 1) reflect a deliberate, conservative identification strategy. These specimens either correspond to putative new species currently undergoing formal description in ongoing revisionary works or belong to species complexes where diagnostic boundaries remain unresolved. Collection data were cleaned and standardised in OpenRefine 3.7.2 and validated in the GBIF Data Validator and the geographic validation service in QGIS provided by SiB Colombia.

## Geographic coverage

### Description

Sampling was carried out on the northern and western slopes of the Sierra Nevada de Santa Marta, specifically in the basins of the Fundación and Don Diego Rivers, covering different altitudinal ranges (0–2,000 m a.s.l.) (Fig. 1). Coordinate ranges for each sampled locality are given in Table 1.

## Taxonomic coverage

### Description

Beetles of the families Curculionidae (Fig. 2), Passalidae (Fig. 3) and Scarabaeidae (Scarabaeinae, Fig. 4) were identified to the lowest taxonomic level possible.

### Taxa included

**Table:** 

Rank	Scientific Name	Common Name
family	Curculionidae	Weevils
family	Passalidae	Bess beetles
family	Scarabaeidae	Scarab beetles
subfamily	Scarabaeinae	dung beetles

## Temporal coverage

**Data range:** 2022-6-13 – 2023-10-12.

### Notes

Sampling was primarily conducted between 13-06-2022 and 07-12-2022; a supplementary sampling event was carried out on 12-10-2023.

## Collection data

### Collection name

Colección Entomológica Universidad del Magdalena.

### Collection identifier

https://www.gbif.org/grscicoll/collection/a03172d2-ea56-439c-a71d-86235e80c72e

## Usage licence

### Usage licence

Other

### IP rights notes

CC BY 4.0

## Data resources

### Data package title

Beetles (Insecta, Coleoptera) from the Sierra Nevada de Santa Marta, deposited in the Centro de Colecciones Biológicas of the Universidad del Magdalena

### Resource link

https://doi.org/10.5281/zenodo.20725189

### Number of data sets

3

### Data set 1.

#### Data set name

Curculionidae from the Sierra Nevada de Santa Marta, deposited in the Centro de Colecciones Biológicas of the Universidad del Magdalena

#### Data format

Dataset

#### Download URL

https://doi.org/10.5281/zenodo.20725189

#### Description

This dataset compiles information from 288 biological records for 666 individuals of Curculionidae. It comprises taxonomic, spatial and curatorial information for dry-mounted and ethanol-preserved specimens collected from localities in the Sierra Nevada de Santa Marta, Colombia (Maestre-Guerra et al. 2026).

### Data set 2.

#### Data set name

Passalidae from the Sierra Nevada de Santa Marta, deposited in the Centro de Colecciones Biológicas of the Universidad del Magdalena

#### Data format

Dataset

#### Download URL

https://doi.org/10.5281/zenodo.20725189

#### Description

This dataset compiles information from 240 biological records corresponding to 1035 individuals of the family Passalidae. It comprises taxonomic, spatial and curatorial information for dry-mounted and ethanol-preserved specimens, collected from localities in the Sierra Nevada de Santa Marta, Colombia (Maestre-Guerra et al. 2026).

### Data set 3.

#### Data set name

Scarabaeinae from the Sierra Nevada de Santa Marta, deposited in the Centro de Colecciones Biológicas of the Universidad del Magdalena

#### Data format

Dataset

#### Download URL

https://doi.org/10.5281/zenodo.20725189

#### Description

This dataset compiles information from 2435 biological records corresponding to 11885 individuals of the subfamily Scarabaeinae. It comprises taxonomic, spatial and curatorial information for dry-mounted and ethanol-preserved specimens collected from localities in the Sierra Nevada de Santa Marta, Colombia (Maestre-Guerra et al. 2026).

The following section presents the Darwin Core attributes and their corresponding descriptions for the three datasets (Curculionidae, Passalidae, and Scarabaeinae) comprising the data package. Unless explicitly stated otherwise, the attributes described apply to all three datasets. (*) The corresponding taxon name identifies attributes exclusive to a particular taxonomic group.

**Data set 3.** 

Column label	Column description
occurrenceID	An identifier for the dwc:Occurrence.
basisOfRecord	The specific nature of the data record.
type	The nature or genre of the resource.
institutionCode	The name (or acronym) in use by the institution having custody of the object(s) or information referred to in the record.
institutionID	An identifier for the institution having custody of the object(s) or information referred to in the record. A constant ("891.780.111-8")
collectionCode	The name, acronym, coden, or initialism identifying the collection or data set from which the record was derived.
collectionID	An identifier for the collection or dataset from which the record was derived. A constant ("https://scientific-collections.gbif.org/collection/a03172d2-ea56-439c-a71d-86235e80c72e")
catalogNumber	An identifier (preferably unique) for the record within the data set or collection.
datasetName	The name identifying the dataset from which the record was derived.
modified	Date on which the resource was changed.
language	A language of the resource. A constant ("es").
recordNumber	An identifier given to the dwc:Occurrence at the time it was recorded.
recordedBy	A list of names of people, groups, or organizations responsible for recording the original dwc:Occurrence.
recordedByID	A list of the globally unique identifier for the person, people, groups, or organizations responsible for recording the original dwc:Occurrence.
individualCount	The number of individuals present at the time of the dwc:Occurrence.
organismRemarks	Comments or notes about the dwc:Organism instance. *Passalidae
lifeStage	The age class or life stage of the dwc:Organism(s) at the time the dwc:Occurrence was recorded.
occurrenceStatus	A statement about the presence or absence of a dwc:Taxon at a dcterms:Location.
preparations	A list of preparations and preservation methods for a dwc:MaterialEntity.
disposition	The current state of a dwc:MaterialEntity with respect to a collection.
occurrenceRemarks	Comments or notes about the dwc:Occurrence. *Curculionidae
samplingProtocol	The names of, references to, or descriptions of the methods or protocols used during a dwc:Event.
eventDate	The date-time or interval during which a dwc:Event occurred. For occurrences, this is the date-time when the dwc:Event was recorded.
year	The four-digit year in which the dwc:Event occurred, according to the Common Era Calendar.
month	The integer month in which the dwc:Event occurred.
day	The integer day of the month on which the dwc:Event occurred.
verbatimEventDate	The verbatim original representation of the date and time information for a dwc:Event.
fieldNotes	One of a) an indicator of the existence of, b) a reference to (publication, URI), or c) the text of notes taken in the field about the dwc:Event.
continent	The name of the continent in which the dcterms:Location occurs.
country	The name of the country or major administrative unit in which the dcterms:Location occurs.
countryCode	The standard code for the country in which the dcterms:Location occurs.
stateProvince	The name of the next smaller administrative region than country (state, province, canton, department, region, etc.) in which the dcterms:Location occurs.
county	The full, unabbreviated name of the next smaller administrative region than stateProvince (county, shire, department, etc.) in which the dcterms:Location occurs.
municipality	The full, unabbreviated name of the next smaller administrative region than county (city, municipality, etc.) in which the dcterms:Location occurs.
locality	The specific description of the place.
minimumElevationInMeters	The lower limit of the range of elevation (altitude, usually above sea level), in meters.
locationRemarks	Comments or notes about the dcterms:Location.
verbatimLatitude	The verbatim original latitude of the dcterms:Location.
verbatimLongitude	The verbatim original longitude of the dcterms:Location.
verbatimCoordinates	The verbatim original spatial coordinates of the dcterms:Location.
verbatimCoordinateSystem	The coordinate format for the dwc:verbatimLatitude and dwc:verbatimLongitude or the dwc:verbatimCoordinates of the dcterms:Location.
verbatimSRS	The spatial reference system (SRS) used for the coordinates recorded in dwc:verbatimLatitude and dwc:verbatimLongitude.
decimalLatitude	The geographic latitude (in decimal degrees, using the spatial reference system given in dwc:geodeticDatum) of the geographic center of a dcterms:Location.
decimalLongitude	The geographic longitude (in decimal degrees, using the spatial reference system given in dwc:geodeticDatum) of the geographic center of a dcterms:Location.
geodeticDatum	The ellipsoid, geodetic datum, or spatial reference system (SRS) upon which the geographic coordinates given in dwc:decimalLatitude and dwc:decimalLongitude are based.
georeferenceSources	A list (concatenated and separated) of maps, gazetteers, or other resources used to georeference the dcterms:Location.
identifiedBy	A list of names of people, groups, or organizations who assigned the dwc:Taxon to the subject.
identifiedByID	A list of the globally unique identifier for the person, people, groups, or organizations responsible for assigning the dwc:Taxon to the subject.
dateIdentified	The date on which the subject was determined as representing the dwc:Taxon.
verbatimIdentification	A string representing the taxonomic identification as it appeared in the original record.
identificationRemarks	Comments or notes about the dwc:Identification. *Curculionidae and Scarabaeinae
identificationQualifier	A controlled value to express the determiner's doubts about the dwc:Identification. *Curculionidae and Scarabaeinae
scientificName	The full scientific name, with authorship and date information if known.
scientificNameAuthorship	The authorship information for the dwc:scientificName formatted according to the conventions of the applicable dwc:nomenclaturalCode.
higherClassification	A list of taxa names terminating at the rank immediately superior to the referenced dwc:Taxon.
kingdom	The full scientific name of the kingdom in which the dwc:Taxon is classified.
phylum	The full scientific name of the phylum or division in which the dwc:Taxon is classified.
class	The full scientific name of the class in which the dwc:Taxon is classified.
order	The full scientific name of the order in which the dwc:Taxon is classified.
superfamily	The full scientific name of the superfamily in which the dwc:Taxon is classified.
family	The full scientific name of the family in which the dwc:Taxon is classified.
subfamily	The full scientific name of the subfamily in which the dwc:Taxon is classified.
tribe	The full scientific name of the tribe in which the dwc:Taxon is classified.
subtribe	The full scientific name of the subtribe in which the dwc:Taxon is classified. *Scarabaeinae
genus	The full scientific name of the genus in which the dwc:Taxon is classified.
subgenus	The full scientific name of the subgenus in which the dwc:Taxon is classified.
specificEpithet	The name of the first or species epithet of the dwc:scientificName.
infraspecificEpithet	The name of the lowest or terminal infraspecific of the dwc:scientificName. *Scarabaeinae
taxonRank	The taxonomic rank of the most specific name in the dwc:scientificName.
verbatimTaxonRank	The taxonomic rank of the most specific name in the dwc:scientificName as it appears in the original record. *Curculionidae and Scarabaeinae
nomenclaturalCode	The nomenclatural code under which the dwc:scientificName is constructed.

## Figures and Tables

**Figure 1.**
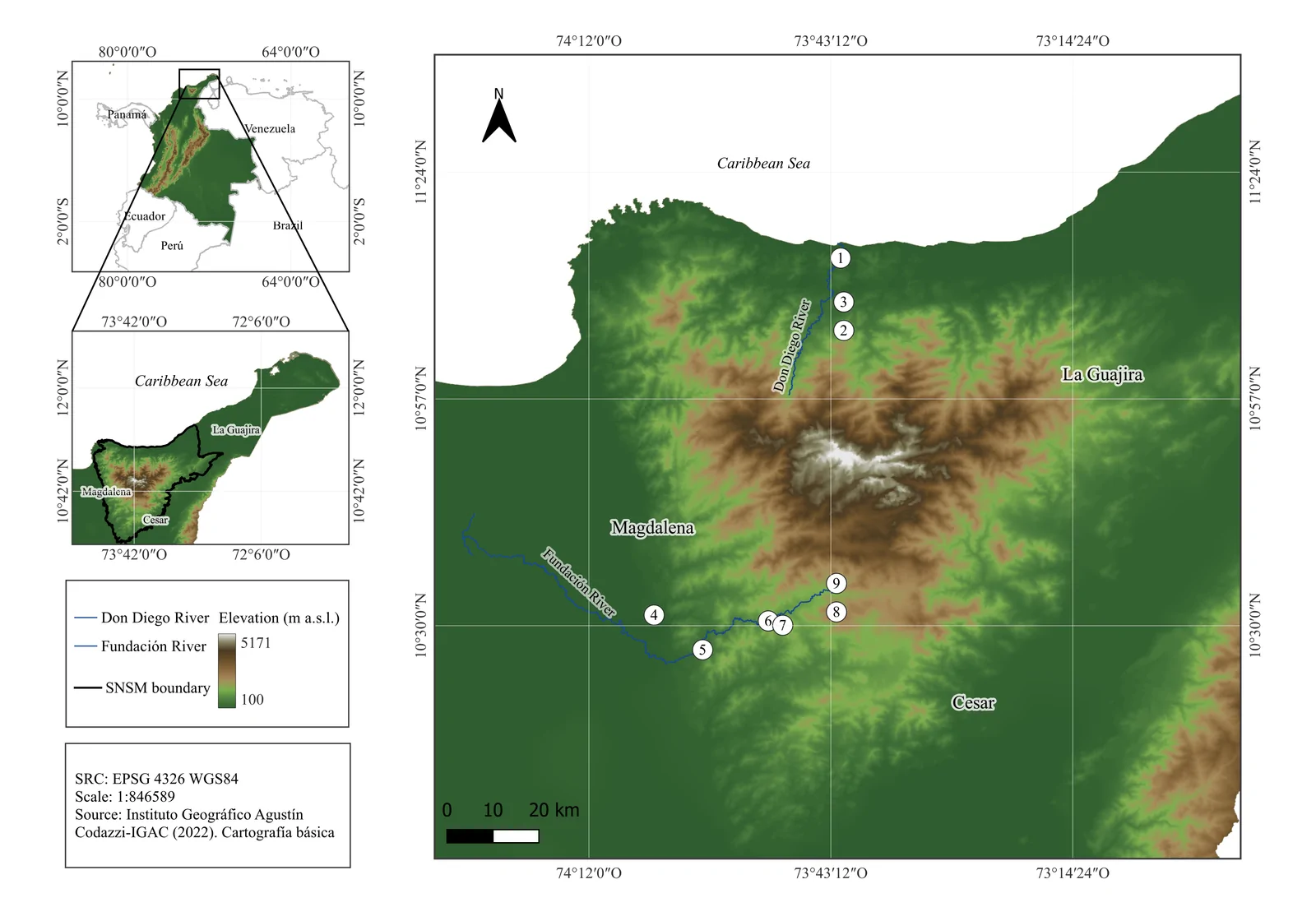
Sampled localities for Curculionidae, Passalidae, Scarabaeinae in two rivers of the Sierra Nevada de Santa Marta, Colombia. Northern slope, Don Diego river; southwest slope Fundación river. Numbers indicate sampling stations from low land to high land.

**Figure 2.**
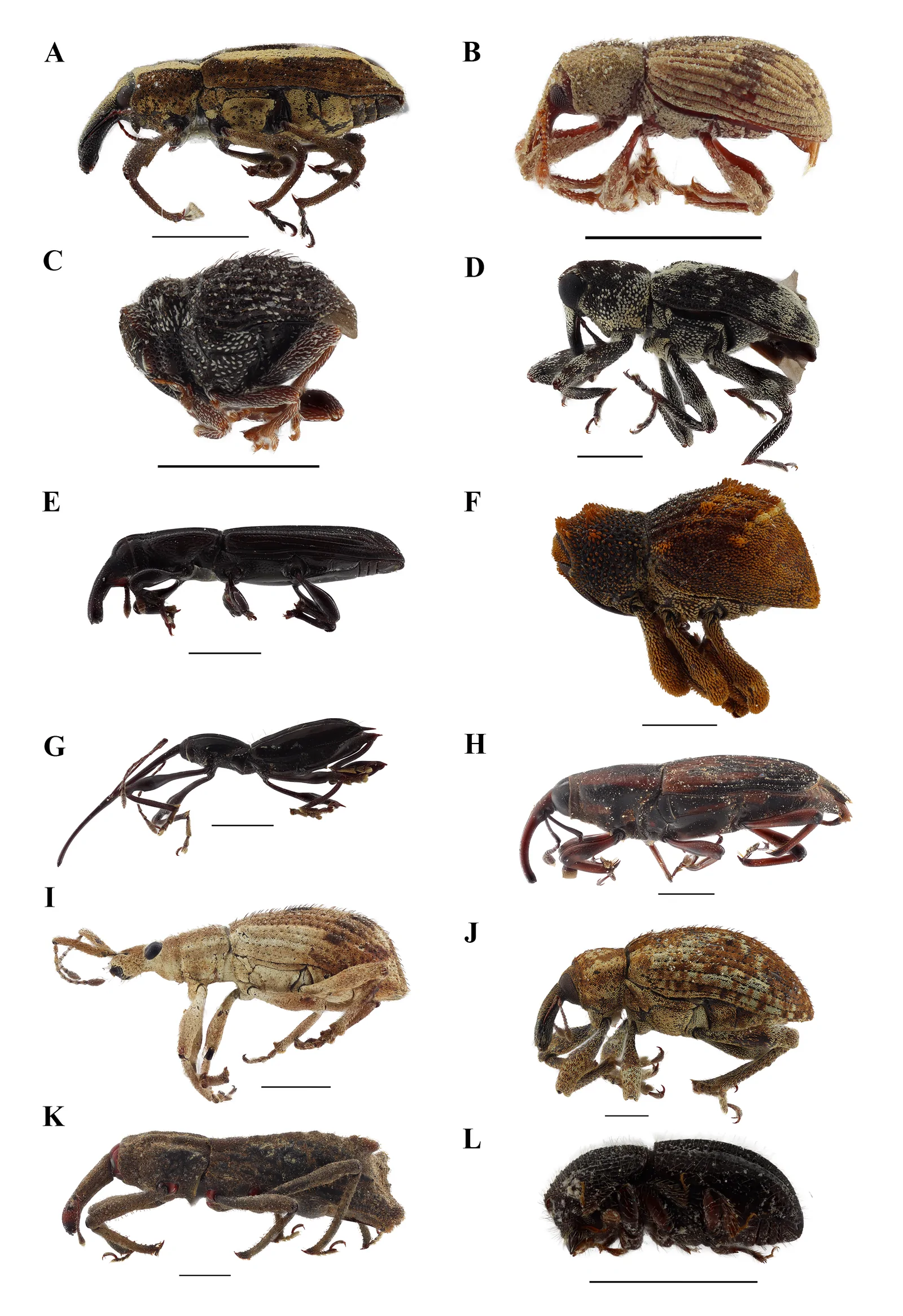
Curculionidae subfamilies recorded in the study area: **A** Baridinae; **B** Brachycerinae; **C** Ceutorhynchinae; **D** Conoderinae; **E** Cossoninae; **F** Cryptorhynchinae; **G** Curculioninae; **H** Dryophthorinae; **I** Entiminae; **J** Hyperinae; **K** Molytinae; **L** Scolytinae. Scale bars: 1.0 mm (**B–F**, **J**, **L**); 2.0 mm (**A**, **G–I**, **K**).

**Figure 3.**
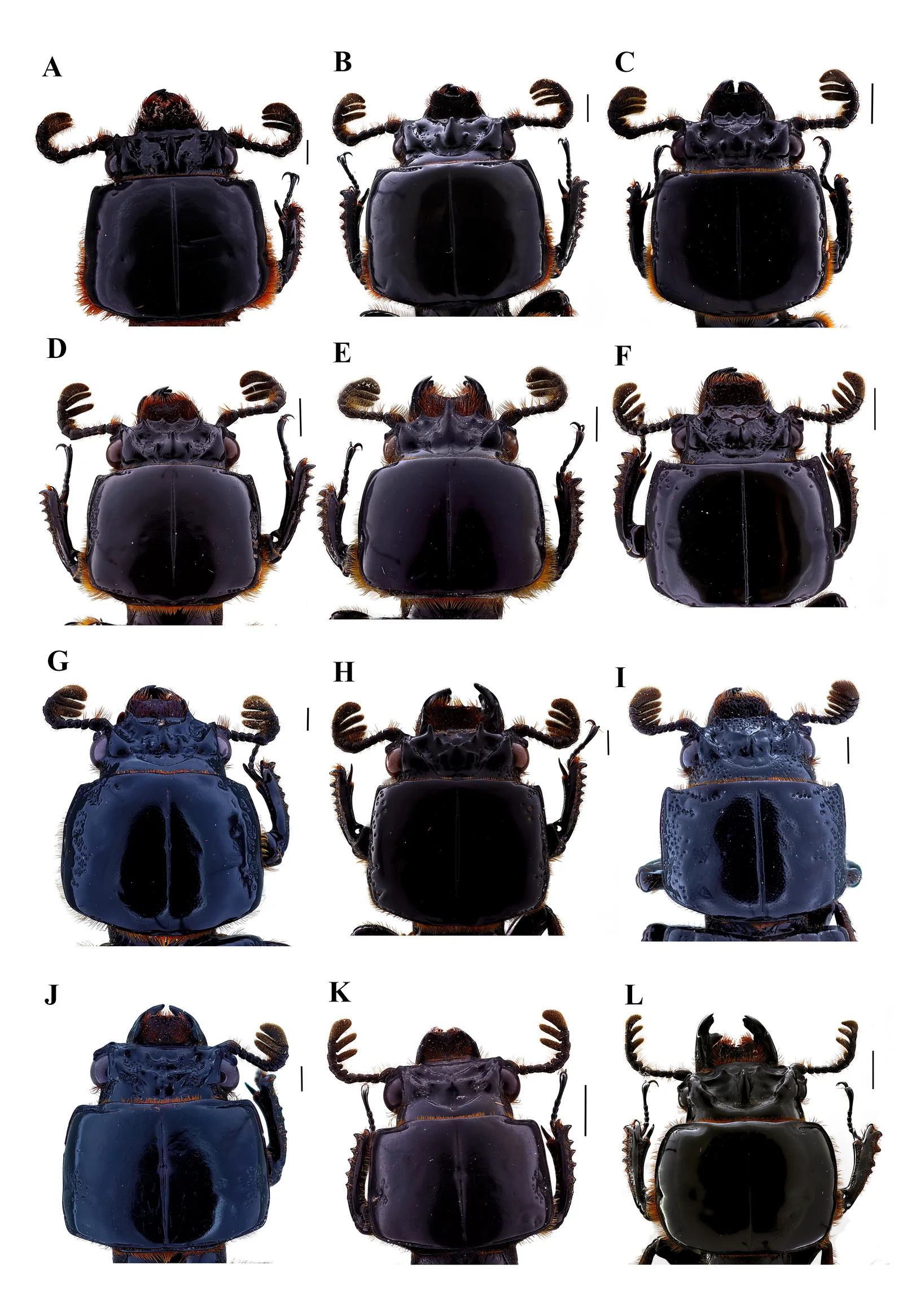
Passalidae species recorded in the study area: **A** Passalus (Passalus) coniferus Eschscholtz, 1829; **B** Passalus (Passalus) interruptus (Linneus, 1758); **C** *Passalus (Passalus) interstitialis E*schscholtz, 1829; **D** Passalus (Passalus) punctiger Lepeletier & Serville, 1825; **E** Passalus (Passalus) serankuai Jiménez-Ferbans, Reyes-Castillo & Amat-García, 2014; **F** Passalus (Pertinax) punctatostriatus Percheron, 1835; **G** Passalus (Pertinax) unimagdalenae Jiménez-Ferbans, Reyes-Castillo & Amat-García, 2012; **H** *Paxillus leachi* MacLeay, 1819; **I** *Spasalus crenatus* (MacLeay, 1819); **J** *Popilius erotylus* Reyes-Castillo & Castillo, 1992; **K** *Popilius marginatus* (Percheron, 1835); **L** Veturius (Publius) impressus Hincks, 1852. Scale bars: 1.0 mm (**G–J**); 2.0 mm (**A**, **C–F**, **K**); 3.0 mm (**B**); 4.0 mm (**L**).

**Figure 4.**
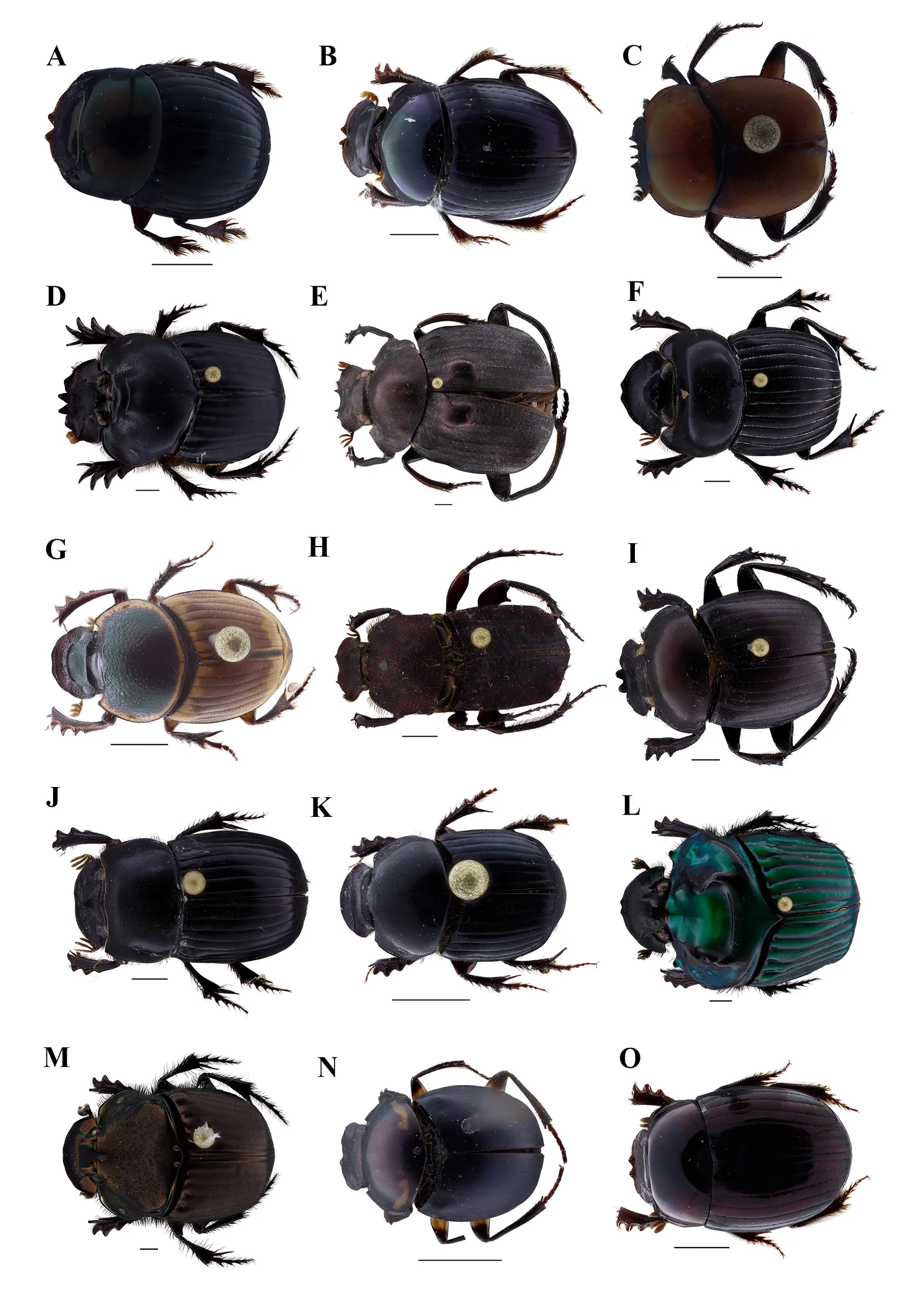
Scarabaeinae genera recorded in the study area: **A** *Ateuchus*; **B** *Canthidium*; **C** *Canthon*; **D** *Coprophanaeus*; **E** *Deltochilum*; **F** *Dichotomius*; **G** *Digitonthophagus*; **H** *Eurysternus*; **I** *Malagoniella*; **J** *Ontherus*; **K** *Onthophagus*; **L** *Oxysternon*; **M** *Phanaeus*; **N** *Scybalocanthon*; **O** *Uroxys*. Scale bars: 1.0 mm (**A**, **B**, **M**, **O**); 2.0 mm (**C–L**, **N**).

**Table 1.** Geographical coordinates for the sampled polygon at each locality.

**River**	**Locality**	**Coordinate range**
Fundación	Finca Villa Lorena (100 m)	10°30'55'' - 10°31'20''N; 74°03'59'' - 74°03'58''W
	Kankawarwa (300–400 m)	10°26'49'' - 10°27'08''N; 73°58'09''- 73°58'27''W
	Umake (800 m)	10°30'05'' - 10°30'37''N; 73°49'26'' - 73°50'58''W
	Umake (1200 m)	10°29'57'' - 10°30'20''N; 73°48'49'' - 73°49'32''W
	Ko’chukwa (1600 m)	10°33'17'' - 10°35'37''N; 73°42'02'' - 73°44'05''W
	Ko’chukwa (2000 m)	10°32'53'' - 10°33'17''N; 73°42'51'' - 73°43'02''W
Don Diego	Finca Tafur (30 m)	11°13'10'' - 11°13'51''N; 73°41'27'' - 73°42'01''W
	Bunkwimake (400 m)	11°06'46'' - 11°07'29''N; 73°41'39'' - 73°42'13''W
	Bunkwimake (800 m)	11°06'18'' - 11°07'11"N; 73°41'09'' - 73°41'44''W
